# Laparoscopic-assisted ERCP is a safe procedure with good outcomes: experience from a single high-volume upper GI unit

**DOI:** 10.1308/rcsann.2026.0052

**Published:** 2026-07-02

**Authors:** L Kenny, C Strong, S Bonnington, EJ Nevins

**Affiliations:** ^1^ Northumbria Healthcare NHS Foundation Trust, UK; ^2^ South Tyneside and Sunderland NHS Foundation Trust, UK

**Keywords:** Cholangiopancreatography, Endoscopic retrograde, Laparoscopy, Choledocholithiasis, Gastric bypass

## Abstract

**Introduction:**

Patients who have undergone Roux-en-Y gastric bypass (RYGB) no longer have anatomy that is amenable to trans-oral endoscopic retrograde cholangio-pancreatogram (ERCP). Laparoscopic-assisted ERCP (LA-ERCP), which combines laparoscopic and endoscopic techniques to access the patient’s biliary tree, is a possible solution. Historically, there have been concerns about the safety and feasibility of this procedure. The aim of this case series is to explore the outcomes of LA-ERCP at a single high-volume centre.

**Methods:**

This is a retrospective case series of all patients undergoing LA-ERCP at a single high-volume UK centre between 2015 and 2024.

**Results:**

A total of 17 patients underwent 19 LA-ERCPs during the above time period. Of the 19 LA-ERCPs, 16 were completed successfully with no complications and the majority of patients were discharged home within four days post-procedure. This is the largest reported UK cohort of patients who have undergone LA-ERCP.

**Conclusion:**

LA-ERCP is an effective, safe and viable option for patients who are unable to undergo trans-oral ERCP.

## Introduction

Endoscopic retrograde cholangio-pancreatogram (ERCP) involves passing an endoscope into the second part of the duodenum to access the biliary tree. It is widely recognised as a standard treatment in patients who have symptomatic choledocholithiasis.^1,2^

Trans-oral ERCP is only possible if the patient’s duodenum is accessible. In patients who have had upper gastrointestinal (GI) reconstruction or have an impassable oesophagus or stomach due to other pathology, this is not always the case.

Metabolic and bariatric surgery is an expanding specialty as the prevalence of obesity and of obesity-related illness continues to rise worldwide. Roux-en-Y gastric bypass (RYGB) is a surgical option to manage obesity. It involves dividing the stomach into a gastric pouch, with an alimentary limb of jejunum then being anastomosed onto this pouch. A second anastomosis is performed to allow the gastric remnant and biliopancreatic limb to drain into the common channel (Figure 1).

**Figure 1:**
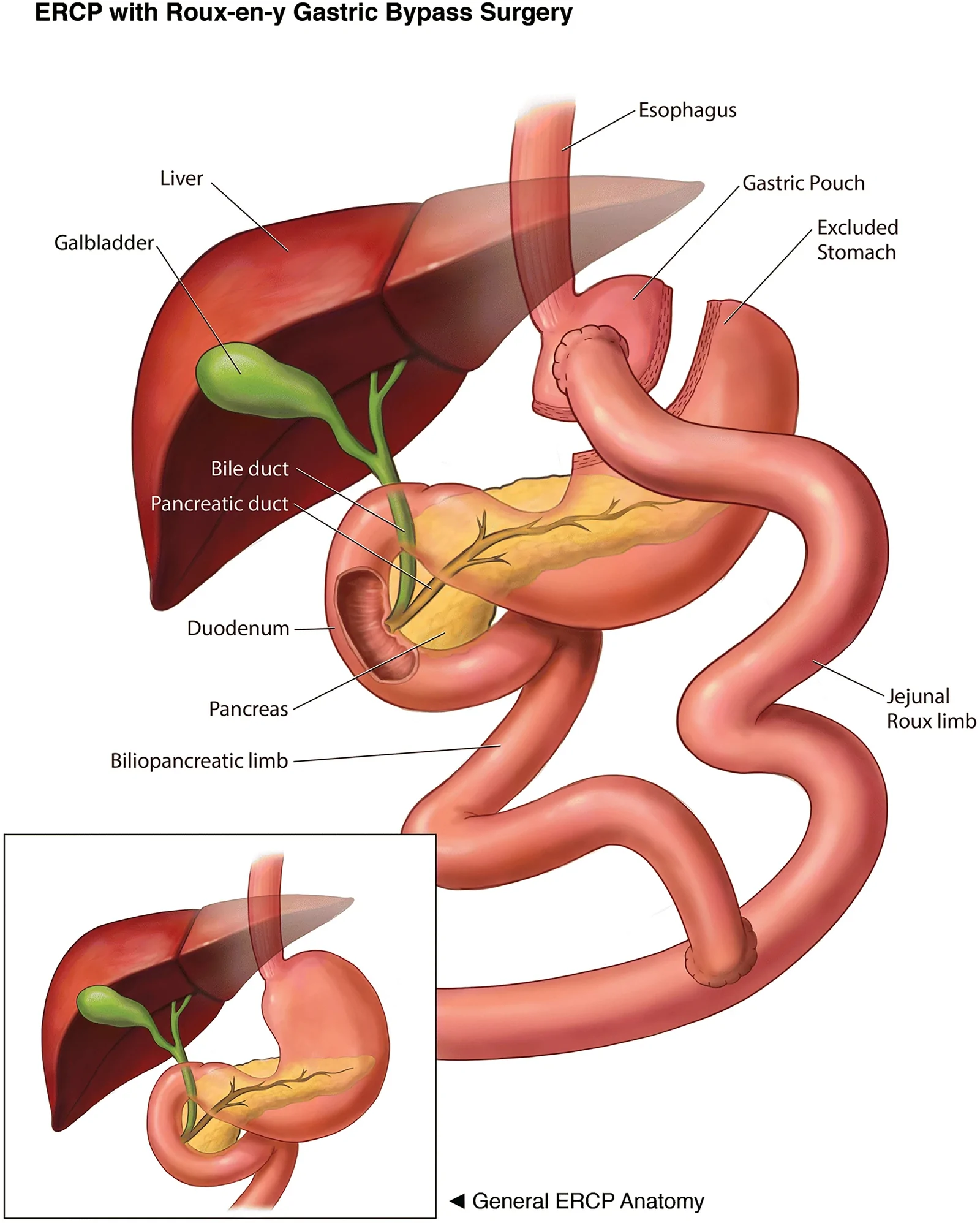
Gastric anatomy post RYGB (standard anatomy in bottom left).^3^ RYGB = Roux-en-Y gastric bypass

In patients who have had RYGB, a standard trans-oral ERCP is not possible. A number of endoscopic options have been proposed to manage this. These include laparoscopic-assisted transgastric ERCP (LA-ERCP), double-balloon ERCP, or endoscopic ultrasound directed transgastric ERCP (EDGE). Another option involves carrying out a laparoscopic common bile duct exploration at the same time as a laparoscopic cholecystectomy.

The authors recommend that if the patient’s gallbladder is still in situ, and there is expertise to perform laparoscopic bile duct exploration and cholecystectomy in a single sitting, this represents optimum management. However, bile duct exploration can be challenging if a cholecystectomy has already been performed or if the bile duct is too small to facilitate choledochoscopy; choledochotomy in a narrow bile duct can also lead to bile duct stricture.

EDGE is becoming increasingly widespread practice in recent years. It is usually a two-stage procedure with around two weeks between stages, although single-stage EDGE is possible. EDGE involves using endoscopic ultrasound to form a gastro-gastric fistula between the gastric pouch and the gastric remnant, allowing the endoscopist to then carry out a trans-oral ERCP through this fistula (Figure 2). The gastro-gastric fistula is intended to be temporary, but there are reported cases in which this has persisted and can lead to weight gain. In one UK case-series, 1 of the 14 patients undergoing EDGE had a persistent gastro-gastric fistula and subsequent weight gain. Therefore, the authors have some concerns that in some cases EDGE may lead to weight regain following RYGB.^4^

**Figure 2:**
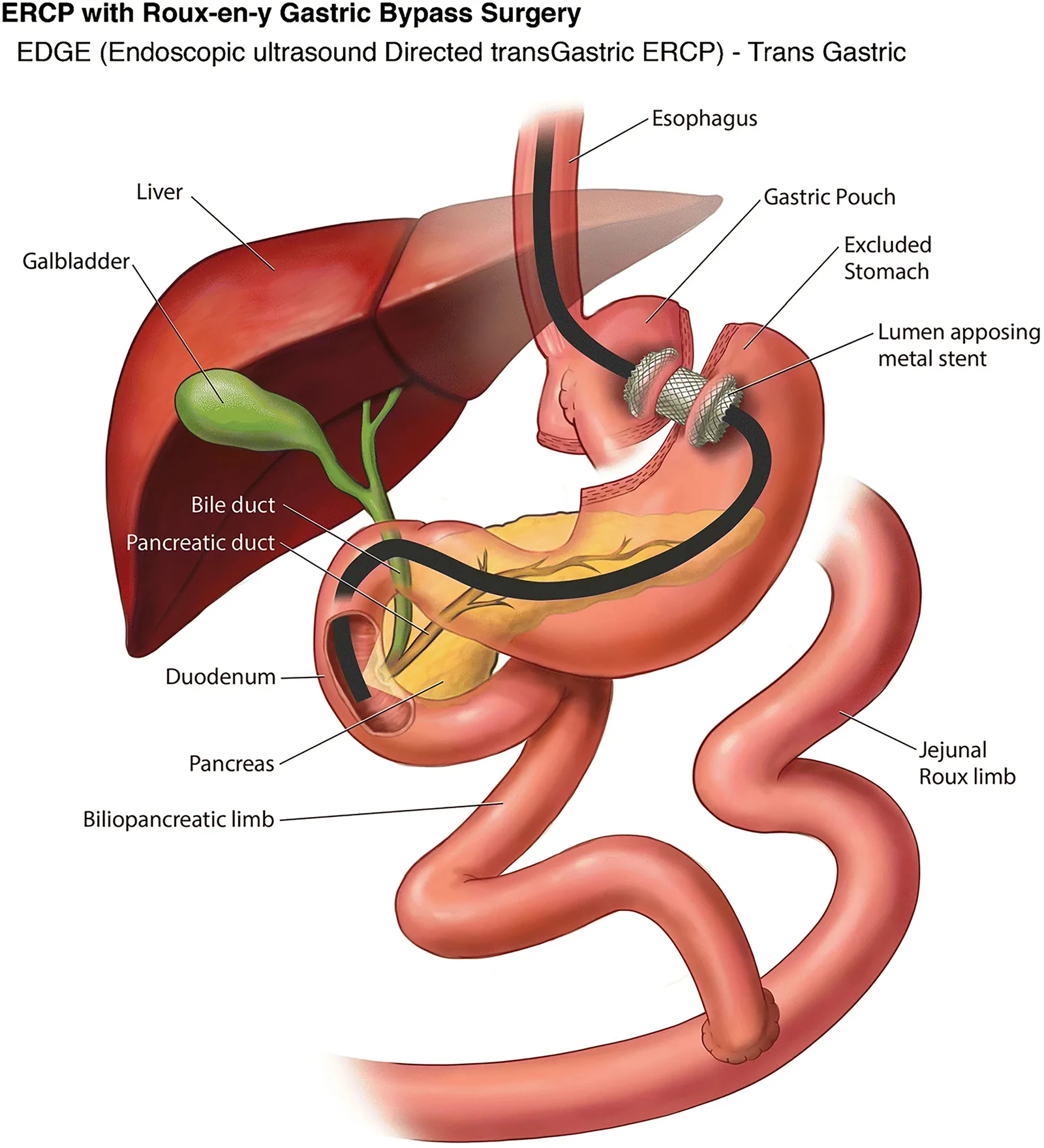
EDGE procedure.^3^ EDGE = endoscopic ultrasound directed transgastric endoscopic retrograde cholangio-pancreatogram

In LA-ERCP, a surgeon inserts a laparoscopic port into the gastric remnant for the endoscopist. They can then carry out an ERCP via the laparoscopic port and the gastric remnant with a standard duodenoscope, without the risk of gastro-gastric fistula and weight regain. The authors therefore recommend that this may be superior in managing patients with common bile duct (CBD) stones following RYGB, without affecting their weight trajectory (Figure 3).

**Figure 3:**
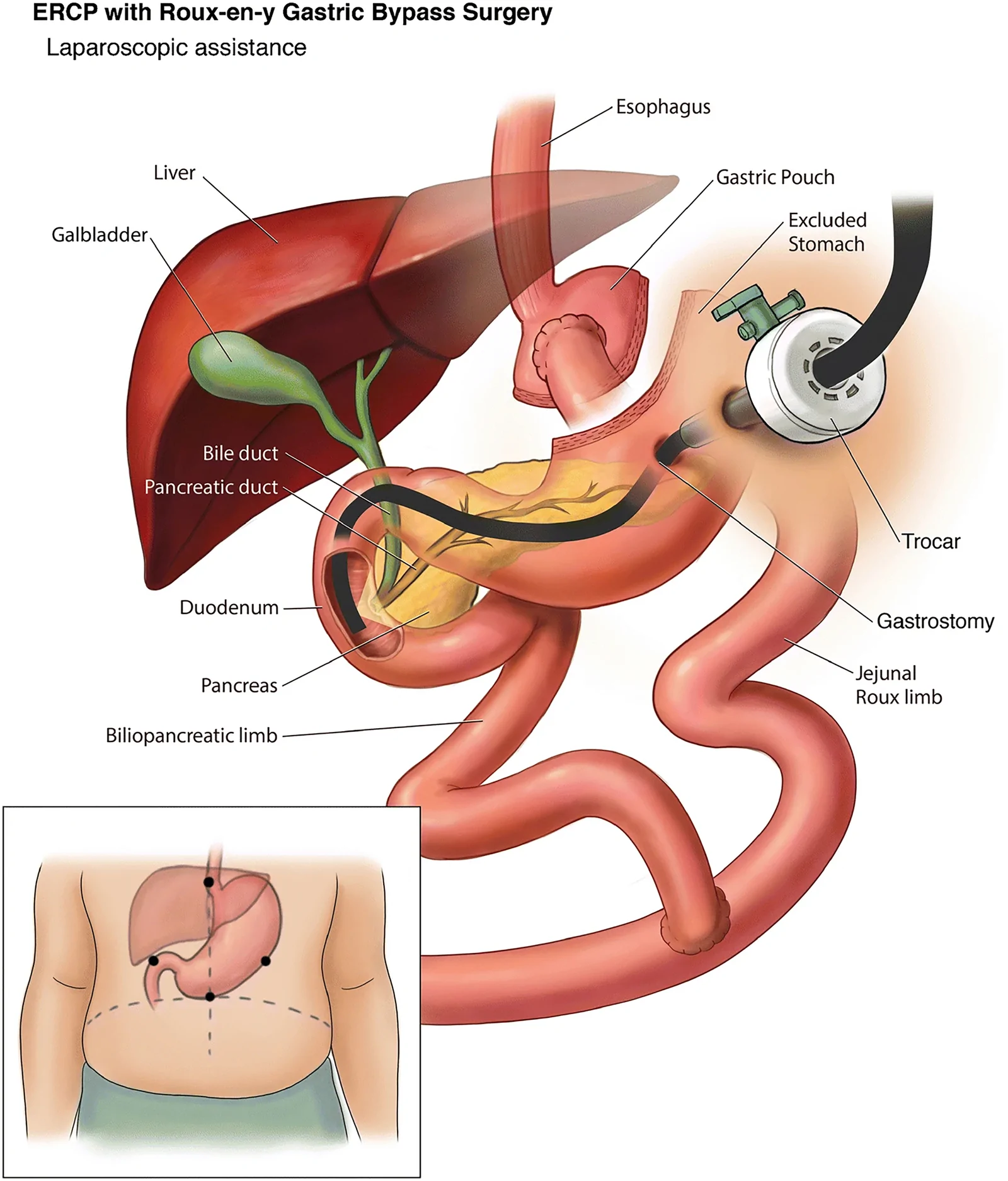
Laparoscopic-assisted ERCP.^3^ ERCP = endoscopic retrograde cholangio-pancreatogram

The aim of this retrospective case series is to illustrate the feasibility and favourable safety profile of LA-ERCP for management of choledocholithiasis in patients for whom trans-oral ERCP is not an option.

## Methods

This is a retrospective case series encompassing all patients undergoing laparoscopic-assisted ERCP from 2014 to 2024 for any reason at Northumbria Healthcare NHS Foundation Trust. The first patient underwent a LA-ERCP in 2015, with the most recent included case in 2024.

Patients were identified using our electronic operation database. A retrospective electronic case note review was performed by a single author. Patient case notes, including operation notes and endoscopy documentation, were reviewed to ensure only patients undergoing LA-ERCP were included. All cases involved both a consultant surgeon and a consultant gastroenterologist.

There are multiple considerations when undertaking LA-ERCP, both in terms of the logistics of the procedure and the technical aspects.

LA-ERCP involves both laparoscopy and ERCP at the same time in the same place, which means that each case involves staff and equipment from multiple specialties that do not routinely work together. A theatre scrub team, an anaesthetic team, an endoscopy team and a surgeon, anaesthetist, radiographer and endoscopist must all be available and in the same room. This can be particularly challenging if the LA-ERCP is taking place as an emergency rather than elective procedure, as is usually the case.

LA-ERCP necessitates specialist equipment including a laparoscopic stack and screen and an endoscopic stack and screen, and fluoroscopy screening, in addition to the surgical and anaesthetic equipment usually present in theatre. It also involves a mix of sterile equipment, involved in the laparoscopy, and nonsterile equipment involved in the ERCP stage of the procedure. The layout of the operating theatre must be carefully considered to accommodate the large number of staff and the mix of equipment. An example layout is shown in Figure 4.

**Figure 4:**
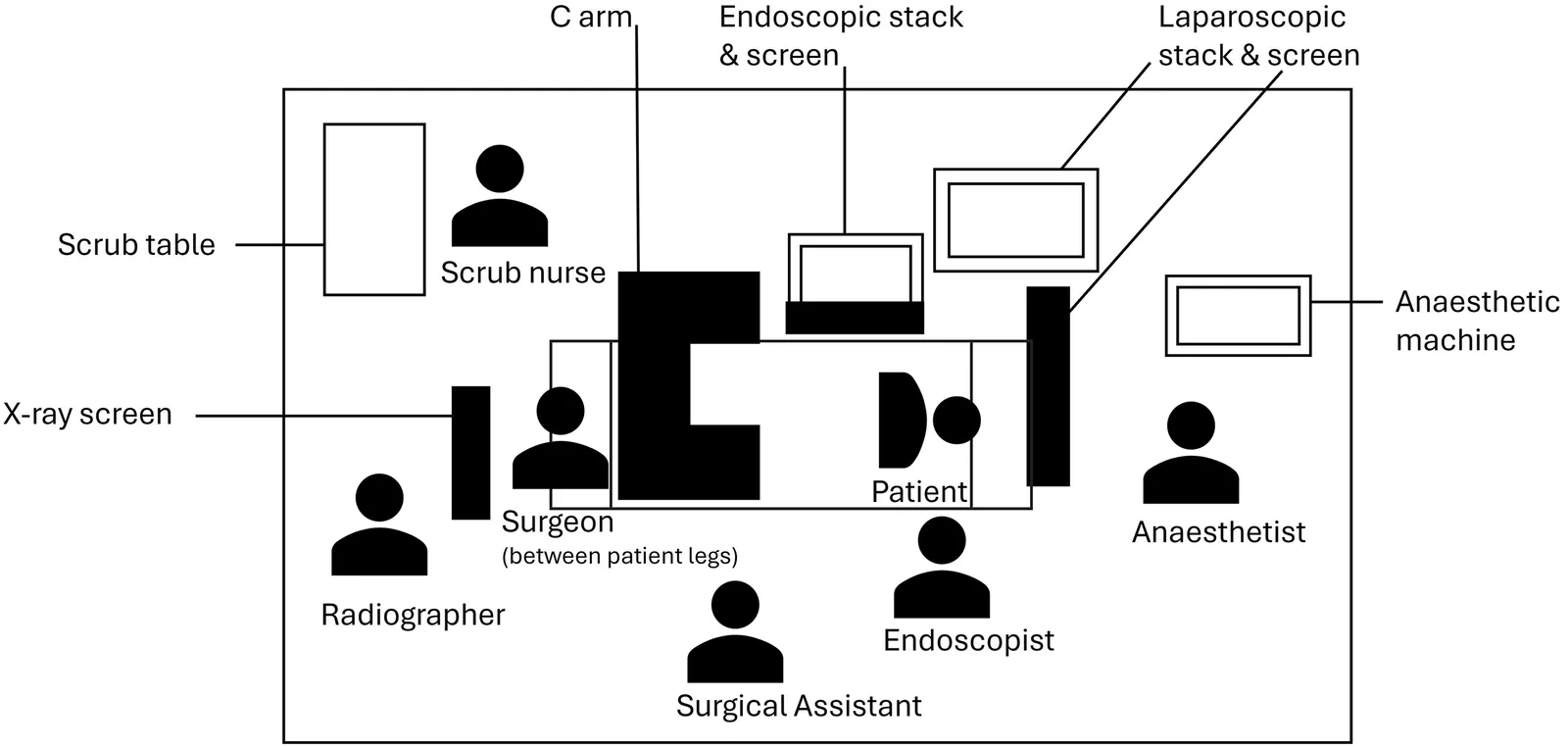
Suggested operating theatre layout.

With regard to the procedure itself, our proposed operating theatre set up is as in Figure 4. The authors recommend positioning the patient in the bariatric position (head up, arms out, sat up). The surgeon operates between the patient’s legs and the surgical assistant stands on the patient’s right side. The anaesthetists and anaesthetic machine are at the patients’ head, with the screen from the laparoscopic stack over the patient’s face. The scrub team and scrub table are positioned by the patient’s right foot, with the ERCP stack at the right shoulder and the C-arm of the X-ray machine coming in from the patient’s right side. This enables the endoscopist to stand at the patient’s left shoulder to access the gastric remnant with a standard duodenoscope.

The surgeon uses their preferred technique to obtain pneumoperitoneum. The surgeon should then identify the gastric remnant and identify a site for gastrotomy. The authors recommend forming a gastrotomy of sufficient size to accommodate a 15mm laparoscopic port.

The ideal position for the gastrotomy is lateral to the gastrojejunal anastomosis, and as close to the gastric fundus as possible, although the costal margin and the presence of adhesions can limit access at this point. When forming the gastrotomy, ideally the positioning of it should allow for the laparoscopic port, the gastrotomy, and the pylorus to be in alignment, which makes intubating the pylorus with the side-viewing ERCP scope less challenging for the endoscopist. If it is difficult to identify the fundus (for example, if the left upper quadrant is hostile), then the gastrotomy can be made lower in the stomach along the greater curve. Alternative access points include medial to the gastrojejunal anastomosis near the pylorus, but this makes intubating the duodenum during ERCP more challenging if the gastrotomy is very close. If the gastrotomy is too low on the stomach then the articulating segment of the ERCP gets caught in the laparoscopic port, which impedes the endoscopist’s ability to navigate the curve of the duodenum.

Once the gastrotomy is created, a purse-string suture is placed around the gastrotomy; this should be full-thickness on the gastric remnant to control any bleeding from the mucosa. The two tails of the purse-string should be inferior to the gastrotomy – allowing the surgeon to manipulate these to create an airtight seal during the procedure.

It is helpful to reduce the intra-abdominal pressures at this point to reduce the space between stomach and abdominal wall. The surgeon should not manipulate the purse-string at this time as this will reduce the size of the gastrotomy. A 15mm port is placed into the gastrotomy and the purse string is pulled tight to create an airtight seal to allow for insufflation of the gastric remnant.

The endoscopist can then carry out an ERCP with a standard scope via the laparoscopic port in the gastric remnant.

Once the duodenoscope is introduced via the 15mm port, the gastric remnant is accessed and ERCP can proceed in a similar fashion to trans-oral ERCP. Access to the duodenum and to the ampulla is generally achieved with a shorter length of scope shaft in the GI tract. This can have implications for scope handling and the manoeuvres required to manipulate the scope tip. Particular care should be taken during sphincterotomy, as manipulation of the scope may cause greater than anticipated movement of the scope tip. Consideration must also be given to the supine position of the patient and the implications for fluoroscopy imaging.

Additionally, gastric insufflation during ERCP leads to dilation of the small bowel, which can cause technical challenges when closing the gastrotomy. Carbon dioxide is used, which reabsorbs relatively quickly, albeit in hours rather than in minutes. As much gas as possible is suctioned out post-procedure but gas travels along the small bowel, meaning that the distal small bowel will be distended, potentially affecting the space available for laparoscopic suturing.

Once the ERCP is concluded, the surgeon closes the gastrotomy; we recommend using a continuous suture, but a linear stapler can be used to close the gastrotomy if the surgeon is less experienced at intracorporeal suturing. The laparoscopic ports can be removed under direct vision, insufflation ceased and the port sites closed in the usual fashion. The authors do not recommend the use of drains unless there are concerns about the integrity of the gastrotomy closure; if so, an omental patch is preferable.

## Results

A total of 17 patients who had undergone 19 LA-ERCPs were identified. Of these 17 patients, 13 had a previous RYGB, 1 had a previous partial gastrectomy and 3 had oesophageal strictures. No patients were excluded; 11 of the patients were female, 6 male. The median age of our patient group was 65 (40–82) years. No patients were ASA 1, eight were ASA 2, seven were ASA 3 and two were ASA 4 (Table 1).

**Table 1:** Summary of patient demographics and procedure outcomes

Median age	65 (40–82) years
Gender	
F	11
M	6
ASA	
1	0
2	8
3	7
4	2
Reason for LA-ERCP	
RYGB	13
Oesophageal stricture	3
Partial gastrectomy	1
Procedure urgency	
Elective	5
Semi-elective	1
Emergency	13
Procedure success	17 of 19
Median length of stay	2 (0–36) days
Complications	
Bleeding (required transfusion)	1
30-day mortality	0

EDGE = endoscopic ultrasound directed transgastric ERCP; ERCP = endoscopic retrograde cholangio-pancreatogram; LA-ERCP = laparoscopic-assisted ERCP; RYGB = Roux-en-Y gastric bypass

Of the procedures performed, 6 were elective or semi-elective, and 13 were emergency. The median time to an emergency LA-ERCP was 3 days (range 1–21 days). The median time to an elective or semi-elective LA-ERCP was longer, at 28.5 days (range 3–119 days).

A total of 17 of 19 LA-ERCPs were completed successfully. One patient had an unsuccessful LA-ERCP, with the endoscopist able to cannulate the CBD but not able to safely carry out a sphincterotomy, and therefore the patient underwent CBD exploration instead. One patient had an attempt at LA-ERCP abandoned due to difficulty accessing the gastric remnant and subsequently had a laparoscopic subtotal cholecystectomy. This was followed by a repeated attempt at LA-ERCP during the same illness episode by a second surgeon. This attempt was successful at clearing their CBD. This highlights the challenges of gastrotomy and surgical access in these patients and highlights the requirements for surgeons to have a full understanding of how to perform this procedure.

A total of 8 of 17 patients had a gallbladder at least partially present at the time of LA-ERCP. Of these eight, six had simultaneous cholecystectomy or completion cholecystectomy during their LA-ERCP. In the eight patients who had a gallbladder present, one had a laparoscopic CBD exploration; this was after an unsuccessful attempt at LA-ERCP. The reasons as to why the remaining seven patients did not undergo CBD exploration are as follows: four of seven had a CBD diameter of <10mm and therefore choledochotomy was felt not to be possible; one of seven had a 10mm duct but a very distal stone and therefore retrieval of this stone with a basket was felt not to be possible; one of seven patients had a high body mass index, therefore the surgeon did not feel they could assess the supraduodenal CBD safely, and one of seven patients had a duct of >10mm but was considered too frail to undergo CBD exploration. Two patients who had a gallbladder present did not undergo simultaneous cholecystectomy at the time of their LA-ERCP. One patient was considered to be too frail for any significant surgery (and therefore had neither a simultaneous cholecystectomy nor a CBD exploration); the other patient who did not undergo simultaneous cholecystectomy had a gallbladder that was felt to be too hostile to remove by the operating surgeon.

One patient was discharged on the day of surgery, and a further seven patients were discharged on the first postoperative day. Four patients were discharged on days two to three post-procedure. Median postprocedural length of stay was 2 days (range 0-36). A total of 16 patients were discharged after returning to a level 1 ward, with 1 patient admitted to the high dependency unit postoperatively for monitoring. There were no conversions to open surgery. There were no unplanned returns to theatre in the three months post-procedure, other than the patient who had an additional attempt at LA-ERCP during their admission.

One patient sustained bleeding from the sphincterotomy and required transfusion. There were no mortalities at 30 days. One patient required a repeat LA-ERCP two years following their original procedure.

## Discussion

This represents the largest reported UK cohort of patients who have undergone LA-ERCP. It demonstrates a favourable safety profile and illustrates that providing this service can be a feasible and effective solution for the management of CBD stones in patients who cannot undergo trans-oral ERCP.

LA-ERCP is relatively resource and personnel intensive. It involves teams from multiple specialties (gastroenterology, endoscopy, radiography) in addition to the surgical, anaesthetic and scrub teams usually present in theatre. It also requires radiographic and endoscopic equipment as well as standard surgical and anaesthetic paraphernalia. This can mean that LA-ERCP is logistically challenging, especially in teams who are less familiar with the required room set up. This cohort demonstrated that there was not a long wait to access LA-ERCP in emergency patients. The current UK literature on LA-ERCP is limited, with only two UK studies in the past ten years. Most recently, a single-centre study in 2020 (*n*=12) demonstrated a high success rate with ten successful LA-ERCPs, and a relatively low complication rate; one patient developed postprocedural pancreatitis and one patient developed chronic pain.^5^ Before this, a smaller single-centre study (*n*=7) showed a 100% success rate in a therapeutic LA-ERCP but noted two minor postprocedural complications: one surgical site infection and one case of post-procedure pancreatitis.^6^

These findings regarding the efficacy of LA-ERCP are supported by a global systematic review, involving 26 publications.^7^ All forms of transgastric ERCP were reviewed, encompassing LA-ERCP, EDGE and other methods such as percutaneous gastrostomy placement. The authors concluded that LA-ERCP appears to be safe and effective, noting a complication rate of 14%.^7^ Of these complications, 80% were related to the gastrotomy formation rather than the ERCP, demonstrating the need to ensure that gastrostomy formation and closure is done in a careful and controlled manner. Post-procedure pancreatitis was the most common ERCP-related complication. However, there were no cases of post-ERCP pancreatitis in the presently reported cohort.

For patients with ductal stones and a gallbladder present, bile duct exploration is our recommended first-line approach for management of choledocholithiasis. A recent European observational study demonstrated that, in patients who have previously had gastric surgery (RYGB, subtotal gastrectomy), laparoscopic common bile duct exploration (LCBDE) at time of laparoscopic cholecystectomy is effective, with 95% of cases achieving ductal clearance.^8^ A complication rate of 8 of 38 (21%) was observed.^8^ The authors recommended LCBDE to be considered over advanced endoscopy techniques, assuming there is surgical experience of this procedure.^8^

When comparing LCBDE directly with LA-ERCP, a large multicentre European study demonstrated similar complication rates between LA-ERCP and LCBDE, noting that LCBDE had a shorter procedural time and generally a shorter hospital stay.^9^ However, LA-ERCP was performed more commonly in the acute setting.

As EDGE has become adopted more widely, comparisons have been drawn increasingly between EDGE and LA-ERCP. Two recent global meta-analyses have reported that LA-ERCP is effective, showing comparable success rates to EDGE and a higher success rate compared with balloon enteroscopy.^10^ However, they reported relatively high complication rates with LA-ERCP at 17% and 20%, respectively.^10,11^ Most of these complications were of mild and moderate severity including pancreatitis, bleeding, wound infection or perforation. This was attributed to the concurrent risk of both gastrotomy formation and ERCP taking place. They reported a post-procedure pancreatitis rate comparable with that seen in trans-oral ERCP. They also reported an 8% (66 of 931) and 7% (67 of 1,213) conversion rate to open surgery, respectively.^10,11^ The present study records a lower complication rate when compared with these previously published meta-analyses, and no conversions to open surgery were required.

The two previously mentioned meta-analyses reported the complication rate of EDGE to be similar to that of LA-ERCP, although the length of stay and procedural time is shorter with EDGE.^10,11^ Alongside the above, a recent meta-analysis focused on comparing EDGE and LA-ERCP directly has demonstrated the same outcomes, namely that EDGE and LA-ERCP are effective, with similar complication rates, but that EDGE has a shorter length of stay and length of procedure.^12^ However, this neglects the fact that EDGE is generally a multistep process, where the patient must undergo multiple invasive procedures. Additionally, concerns about persistence of gastro-gastric fistula and subsequent weight gain post-EDGE were noted.

There is a paucity of evidence about the incidence of fistula persistence and weight gain post-EDGE, possibly due to the lack of long-term follow-up data. Most recently, 1 out of 14 patients in a single-centre UK study experienced persistent gastro-gastric fistula that led to subsequent weight gain.^4^ On review of the global literature around EDGE, the reported rate of persistent gastro-gastric fistula is variable, perhaps reflecting the limited data on this issue. Various publications reported the rate of gastro-gastric fistula persistence as 17%, 31% and 41%.^13–15^ This variability is likely due to some studies having only short follow-up periods. Persistent gastro-gastric fistula is not a complication that occurs after LA-ERCP. This is an important consideration when consenting patients for EDGE, particularly in patients who have undergone RYGB for the treatment of obesity.

## Conclusion

LA-ERCP is a safe and effective method of managing symptomatic gallstones in patients who are unsuitable for trans-oral ERCP, although LCBDE should be considered above LA-ERCP where appropriate. Other options include EDGE, but in patients who have undergone RYGB, EDGE can predispose them to gastro-gastric fistula and weight regain. High-quality randomised evidence is required to identify which is the optimum technique for managing bile duct stones in patients for whom a trans-oral ERCP is not possible.

## Competing interests

The authors declare no competing interests.

## Funding

The author(s) received no financial support for the research, authorship, and/or publication of this article.

## Author contributions

**L Kenny:** Data curation, Formal analysis, Project administration, Writing – original draft, Writing – review & editing. **C Strong:** Supervision, Writing – review & editing. **S Bonnington:** Conceptualisation, Methodology, Supervision, Writing – review & editing. **EJ Nevins:** Conceptualisation, Methodology, Project administration, Supervision, Writing – original draft, Writing – review & editing.

## Artificial Intelligence

The author/s declare that no AI was used to conduct the study or prepare the manuscript.
